# On the Dynamic Electro-Mechanical Failure Behavior of Automotive High-Voltage Busbars Using a Split Hopkinson Pressure Bar

**DOI:** 10.3390/ma14216320

**Published:** 2021-10-22

**Authors:** Tobias Werling, Georg Baumann, Florian Feist, Wolfgang Sinz, Christian Ellersdorfer

**Affiliations:** 1 Mercedes-Benz AG, HPC X631, 71059 Sindelfingen, Germany; 2 VSI—Institute of Vehicle Safety, University of Technology Graz, Inffeldgasse 23/I, 8010 Graz, Austria; florian.feist@tugraz.at (F.F.); wolfgang.sinz@tugraz.at (W.S.); christian.ellersdorfer@tugraz.at (C.E.)

**Keywords:** busbar, split Hopkinson pressure bar, dynamic compression, crashworthiness, insulation failure, battery safety, numerical modeling, thermoplastics

## Abstract

High-voltage busbars are important electrical components in today’s electric vehicle battery systems. Mechanical deformations in the event of a vehicle crash could lead to electrical busbar failure and hazardous situations that pose a threat to people and surroundings. In order to ensure a safe application of busbars, this study investigated their mechanical behavior under high strain rate loading using a split Hopkinson pressure bar. Two different types of high-voltage busbars, consisting of a polyamide 12 and a glass-fiber-reinforced (30%) polyamide 6 insulation layer, were tested. Additionally, the test setup included a 1000 V electrical short circuit measurement to link the electrical with the mechanical failure. It was found that the polyamide 12 insulated busbars’ safety regarding insulation failure increases at high loading speed compared to quasi-static measurements. On the contrary, the fiber-reinforced polyamide 6 insulated busbar revealed highly brittle material behavior leading to reduced bearable loads and intrusions. Finally, the split Hopkinson pressure bar tests were simulated. Existing material models for the thermoplastics were complemented with an optimized generalized incremental stress state-dependent model (GISSMO) with strain rate dependency. A good agreement with the experimental behavior was achieved, although the absence of viscoelasticity in the underlying material models was notable.

## 1. Introduction

Automotive high-voltage busbars (HVBs) play an important role in today’s electric vehicle battery systems. They connect the cell modules inside the battery pack and are used as a power distribution device within the battery system [1]. Depending on the vehicle’s battery system, the HVBs are subjected to electric potentials currently up to 870 V [2] and currents up to 400 A. These, combined with growing battery capacities [3], pose amongst others a challenge in terms of crash safety for researchers and developers. While the safety of batteries and battery cells subjected to internal and external mechanical loading has been investigated [4,5,6,7,8,9,10], to the authors’ knowledge, there have been only a few studies dealing with the mechanical integrity of HVBs under crash loads and its prediction using finite element (FE) models.

Mechanical deformations of the battery pack in the event of a severe vehicle crash can lead to a combination of mechanical, electrical and thermal faults inside the battery. The failure of HVBs in the form of dislocation, rupture or insulation faults can result in electric arcs or short circuits (SCs), which can be a threat to people and surroundings. Ultimately, SCs can lead to a thermal runaway of battery cells [11]. The HVBs’ position within the battery pack enlarges the risk of failure due to a vehicle crash. Commonly placed on the outside or in between the cell modules, intruding objects or other battery components can damage the HVB and its insulation [12].

However, no official regulations exist regarding the mechanical integrity of HVBs so far. Requirements for electrical and thermal performance are derived from high-voltage cable regulations, e.g., [13,14]. Werling et al. [12] investigated the electro-mechanical behavior of HVBs using a quasi-static compression test combined with insulation resistance measurement. The test setup allowed interchangeable indenter geometries and electric potentials of 1000 V. They showed that, depending on the intruding object, the HVB can undergo large plastic deformation. Under compression using a sharp indenter geometry, an SC occurred without complete penetration of the insulation. Their subsequent work [15] treated the development of suitable FE and material models for HVBs. However, the influence of dynamic loading derived from a vehicle crash has not been assessed. Liu et al. [16] performed a dynamic electro-mechanical test with high-voltage cables using a drop tower. They showed that the high-speed loading influenced the critical force as well as the SC occurrence. However, their work was limited to multi-wire cables and electrical potential of 10 V. According to Serban et al. [17] and Siviour et al. [18], there are also plenty of material characterization tests on polymers using a split Hopkinson test bench. However, to the authors’ knowledge, there are no split Hopkinson tests involving a composite structure out of polymer and metal in combination with an electrical potential.

In order to contribute to the safety of HVBs under mechanical loading, this work investigates the dynamic behavior of HVBs under coupled mechanical and electrical loading. The research shall provide critical load limits and knowledge about the HVBs failure behavior under various loading scenarios. To allow accurate and reproducible results, a split Hopkinson Pressure Bar (SHPB) was used in combination with a 1000 V SC measurement. One major advantage of a SHPB over other dynamic testing facilities such as a drop tower test bench is that it allows for an almost constant strain rate over the whole testing procedure [19]. With the high strain rate data obtained through the SHPB experiments, it is possible to further improve existing HVB FE models. These were derived from previous work [15] but only include the quasi-static failure behavior of the busbars.

## 2. Materials and Methods

### 2.1. Materials

**Figure 1:**
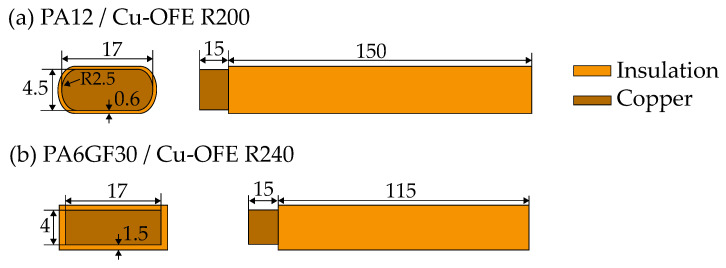
High-voltage busbar geometries (Unit: mm) used in this study: (**a**) PA12/Cu-OFE R200. (**b**) PA6GF30/Cu-OFE R240.

For the experimental work, two different HVB material combinations, which are commonly used, were chosen. The first busbar under investigation consists of a polyamide 12 (PA12) insulation layer with a copper Cu-OFE R200 core. The second busbar is made out of a glass-fiber-reinforced polyamide 6 (PA6GF30) with 30 % glass fiber and copper Cu-OFE R240. While the PA12 busbar was produced in an extrusion process, the PA6GF30 insulated busbar was produced by injection molding. The used thermoplastics are Grilamid XE 3817 (PA12, EMS-GRIVORY, Domat/Ems, Switzerland) and Grilon BG-30-FR (PA6GF30, EMS-GRIVORY, Domat/Ems, Switzerland).The geometries of the busbars are displayed in Figure 1.

In order to obtain comparable results, both busbars were conditioned prior to the experiments using a climate chamber. The accelerated conditioning took place at 70 °C temperature and a relative humidity of 62% according to ISO 1110 [20] until the percentage increase in mass due to water absorption was less than 0.1% in two consecutive measurements. An overview of the materials’ standard properties is given in Table 1. Both HVBs were stripped over a length of 15 mm, to provide a sufficiently large electrical contact area during the experiments (see Figure 1).

### 2.2. Experimental Methods

**Figure 2:**
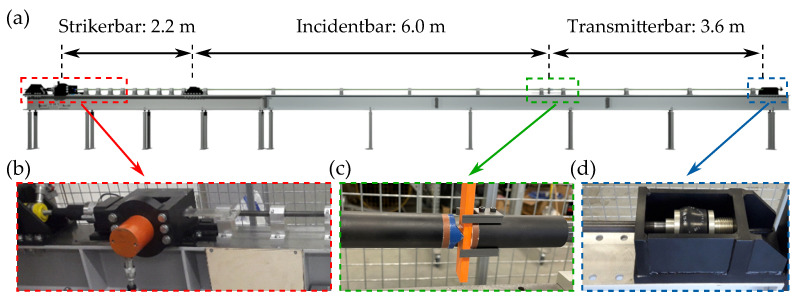
Overview and detailed sections of the split Hopkinson test bench: (**a**) Overview of the SHPB test bench. (**b**) Striker bar with hydraulic and release mechanism to initiate the shock wave. (**c**) Immediate test area with the busbar specimen. (**d**) Damping element in order to absorb excess energy.

The dynamic experiments were conducted using a self made SHPB.This test bench consists of three slender and long aluminum bars (AW 7075) with a diameter of 20 mm. The first bar (striker) is used to store internal, i.e., elastic deformation energy, which is built up through a hydraulic cylinder. The busbar itself is mounted between the second bar (incident) and the third bar (transmitter). By quickly releasing the elastic energy of the striker bar, a one-dimensional shock wave is formed (compare [24]). This shock wave propagates through the striker and incident bar until it reaches the busbar. When the busbar is hit by the shock wave, it is mechanically deformed. During this process, one part of the shock wave travels through the busbar into the transmitter bar while a second part of the wave is reflected back into the incident bar. The shock wave, which is repeatedly traveling through the whole bar system, is gradually attenuated by a damping system at the end of the transmitter bar. An overview of this system is shown in Figure 2.

**Figure 3:**
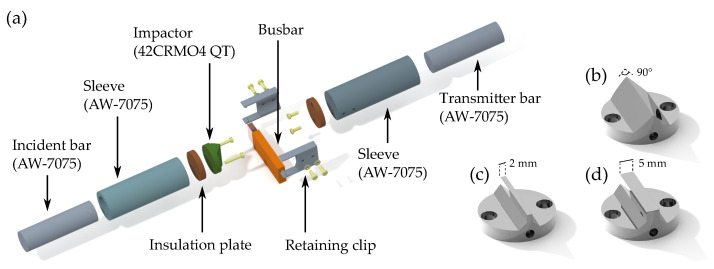
Explosion view of the immediate test area between the incident bar and transmitter bar (**a**), overview of the used impactor shapes V1 (**b**) V5 (**c**) and V4 (**d**).

The SHPB, which is normally used for dynamic, high strain rate, material characterization in tension or compression, was modified to accommodate the HVBs and to allow for rapid exchange of impactors and specimens. The incident side consists of an aluminum sleeve (made of AW-7075), an insulation plate made of Pertinax (an electrically insulating phenoplast) and an exchangeable impactor (made of hardened steel 42CRMO4 QT). The impactor and the insulation plate were screwed to the sleeve using nylon bolts. This was necessary in order to avoid an electric arc from the busbar to the SHPB bars and to the attached strain gauges. Within previous quasi-static characterization tests (see [12]), a total of six different impactor types (V1 to V6) were used. In this study, the three most relevant types (V1, V4 and V5), see Figure 3b–d, respectively, as identified in the previous study, were used. Through retaining clips (made of PLA), the HVBs were held in place and attached to the aluminum sleeve (see Figure 3).

The strains in the incident bar and the transmitter bar due to the shock waves were measured through strain gauges “1-LY13-1.5/120” (HBM, Vienna, Austria). The strain gauge signals were sampled with a rate of 2 MHz using the amplifier “Dewe3-M4” in combination with a “Trion-1820-Multi-4-D-Card” (both Dewetron, Grambach, Austria). In addition to the information from the strain gauges, high-speed videos were also made in order to better analyze the deformation and fracture behavior of the busbars. The sampling rate of the high-speed camera “Phantom Veo 640” (Ametek, Berwyn, IL, USA) was 50,000 fps using a shutter speed of 4 μs. In order to control the pre-load in the striker bar, a load cell “K-K12/N520-G21” with a capacity of 200 kN (Lorenz Messtechnik GmbH, Alfdorf, Germany) was used. The recording of the strain gauges was triggered by an acceleration sensor “Model 1201” with a capacity of up to 1000 g (TE connectivity, Hampton, VA, USA), which was applied on the housing of the release mechanism. The release mechanism includes a brittle failing wear part (compare [24]). When the wear part fails, the stored elastic energy is released in the striker bar. During this process, the wear part segments are smashed against the housing of the release mechanism triggering the acceleration sensor.

**Figure 4:**
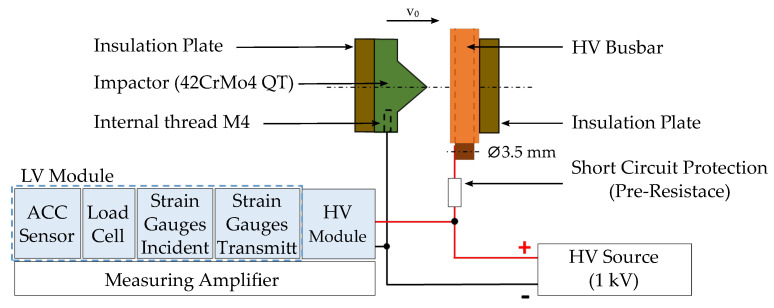
Electrical and mechanical test setup in the immediate vicinity of the test specimen positioned in the SHPB test bench.

The HVB was supplied with 1000 V using a high voltage power unit “HNCs 30,000-4pos” (Heinzinger electronic GmbH, Rosenheim, Germany). The voltage signal was measured with the “Dewe3-M4” (Dewetron, Grambach, Austria) at a sampling rate of 1 MHz. An overview of the measuring concept with the measuring amplifier and power supply is shown in Figure 4.

**Figure 5:**
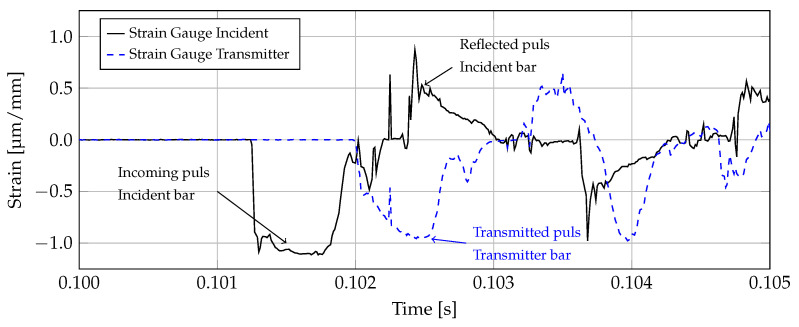
Overview of a typical raw data set from the strain gauges with incoming and reflected pulse in the incident bar (solid black curve) and transmitted pulse in the transmitter bar (dashed blue curve).

The post-processing of the strain gauge signals was carried out using the software “Diadem 2018” (National Instruments, Austin, TX, USA). Figure 5 shows a typical raw data set obtained from the strain gauges on the incident bar and the transmitter bar. The maximum strain deflections of around 1 μm/mm are relatively small to ensure that the bar is loaded purely in the linear elastic range. The incoming strain pulse in the incident bar has a duration of approximately 0.9 ms and determines the maximum time span of the material test. Only the first strain pulse hitting the specimen was considered in the post-processing. Subsequent oscillations, which occur due to reflections in the bars, were disregarded and deemed irrelevant for the evaluations. Due to the fact that the described test setup represents a compression test, both the incoming pulse as well as the transmitted pulse are negative. During reflection, the sign is reversed, which is why the reflected pulse represents a tensile wave.

If the samples show a rather compact geometry (and the material is relatively stiff), the simplified assumption of quasi-static equilibrium is valid. This means that the force on the incident side can be safely assumed to be equal to the force on the transmitter side. In addition, the applicability of the assumption can be validated by summing up the integral of incoming pulse $\epsilon_{I}$ and reflected pulse $\epsilon_{R}$ [25]. If the result equals the integrated transmitted pulse $\epsilon_{T}$, the quasi-static equilibrium can be assumed:

(1) $$\epsilon_{I}+\epsilon_{T}\approx \epsilon_{T}$$

In the present case, there is a difference of about 1.3% between the two sides of the equation. In practice, this value can never be completely zero, because of the thickness of the samples and a finitely high-speed of sound in solids. The offset between the incident and the transmitter side is 2.5 μs for the PA12 Cu-OFE R200 and 2.7 μs for the PA6GF30 Cu-OFE R240 busbar. Due to these rather small deviations, a quasi-static equilibrium can be assumed. Therefore, the force–time characteristic of the busbars can be derived directly from the transmitted pulse.

To convert the measured strain in the transmitter bar $\epsilon_{T}$ to a force *F*, the signal has to be scaled with its structural stiffness (following Chen et al. [25]). The elongation stiffness of the present bar is the Young´s Modulus $E_{B}=72.6$ GPa multiplied with its cross-section $A_{B}=314.2$ mm^2^:

(2) $$F=E_{B}A_{B}\epsilon_{T}$$

Furthermore, the signal was filtered with a Low Pass Butterworth Filter CFC 25,000 and offset. The deformation of the HVBs was evaluated with the help of the high-speed images. Using this method, the intrusion of the impactor is tracked frame by frame until the maximum deformation of the busbar is reached. These pixel-wise movements can be converted into real deformations by scaling them with the help of a known reference length. The achievable resolution at 50,000 fps was 256 × 194 pixels with a traveled distance of the impactor between roughly 35 to 45 pixels depending on the test configuration. Considering a motion tracking error of half the pixel length leads to an overall error of 1.1% to 1.4% for the determination of the total displacement. The generated deformation curve was also filtered and subsequently merged with the calculated force-signal but also with the short circuit signal. In order to gain statistically sound results, each test configuration was carried out with a total of five repetitions.

### 2.3. Numerical Methods

**Figure A1:**
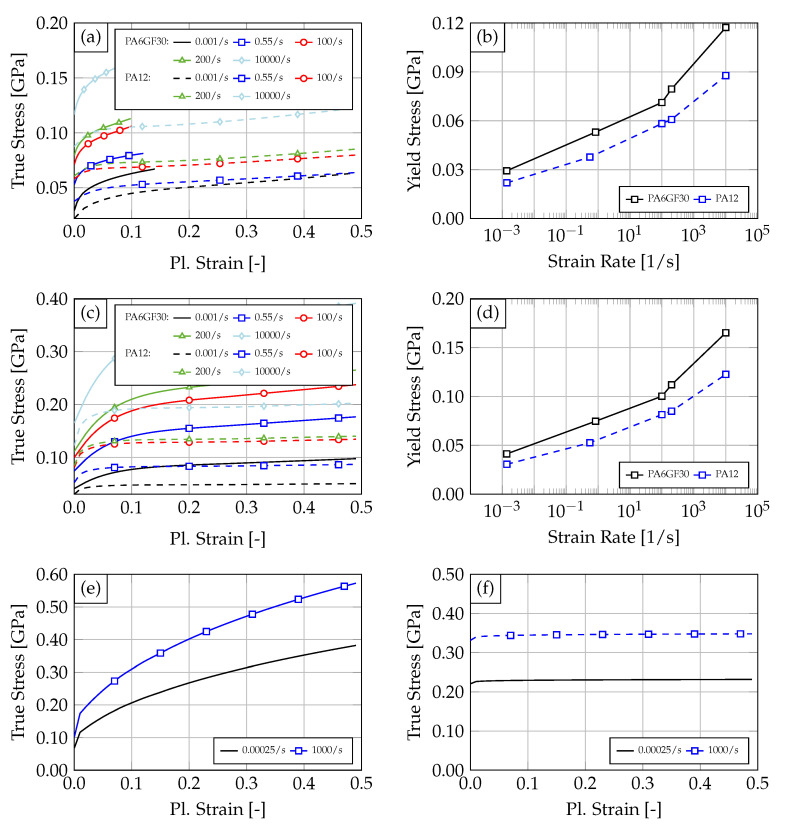
Input parameters of the material models for the HVB insulation and copper-based on [15]: (**a**) Implemented stress–strain curves in tensile direction for PA12 and PA6GF30. (**b**) Strain rate dependent yield stress in tensile direction for PA12 and PA6GF30. (**c**) Implemented stress–strain curves in compressive direction for PA12 and PA6GF30. (**d**) Strain rate dependent yield stress in compressive direction for PA12 and PA6GF30. (**e**) Implemented stress–strain curves in tensile direction for Cu-OFE R200. (**f**) Implemented stress–strain curves in tensile direction for Cu-OFE R240.

In order to allow the prediction of HVB failure under dynamic mechanical loads, a full FE SHPB model was used in combination with the FE solver “LS-DYNA”. The SHPB FE model has been validated using standard material (EN AW1050) coupon tests prior to this investigation. The HVB models and the associated material models were derived from the work of Werling et al. [15]. While the insulation materials were modeled using Mat-124 with tension–compression anisotropy, Mat-24 (piecewise linear plasticity) was used for the copper core. The input material parameters are shown in Appendix A Figure A1. The contact between the insulation and the copper was treated using a penalty-based non-automatic surface-to-surface tiebreak contact. For PA12, a sliding-only option (contact friction μ=0.2) allowing only the interfacial sliding of nodes was chosen [15]. A tiebreak contact with failure option was set for the PA6GF30 insulated busbar. Here, failure of the contact occurs when the contact shear stress reaches a critical limit of τ=33 MPa [15]. After failure, the contact behaves like a standard surface-to-surface contact. A detailed description of the contact treatment can be found in [26].

**Figure 6:**
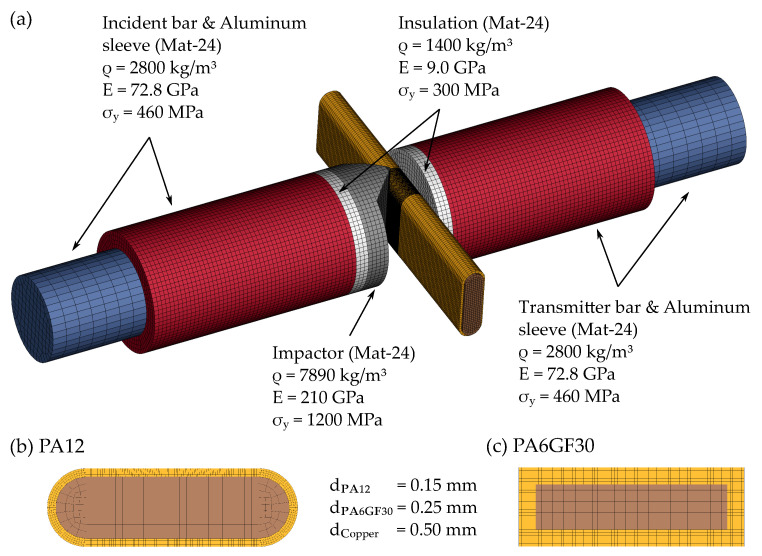
Numerical FE model of the SHPB environment and the HVB: (**a**) Shows the SHPB model in the area of the specimen including the used material models and parameters. (**b**,**c**) Show the cross-section of the impacted HVBs (PA12 and PA6GF30) with the according element sizes.

An extract of the SHPB FE model environment including the HVB is shown in Figure 6a. As the SHPB components are not loaded beyond elastic limits, standard Mat-24 material models were used with the parameters given. In order to simplify the model, the screw fittings between the incident bar (transmitter bar) and the aluminum sleeve as well as between the insulation plate and the aluminum sleeve were modeled as tied connections. For the contact between the impactor and the HVB, an eroding-single-surface contact was used, while the contact between the HVB and the insulation plate was modeled using an automatic-single-surface contact. Frictional parameters were set to μ=0.2.

The GISSMO damage model (generalized incremental stress-state-dependent model) [27] was used in addition to the material models to incorporate the possibility of insulation failure in the FE model. GISSMO was originally developed for sheet metals but has since also been used for the modeling of failure in thermoplastics [28,29]. The phenomenological model enables an incremental path-dependent treatment of material instability and non-linearity [30,31] and the definition of stress triaxiality-dependent failure and instability parameters. The damage evolution is defined by

(3) $$D=\sum {\left(\frac{\epsilon_{p}}{\epsilon_{f}\left(\eta \right)}\right)}^{n}\;\mathrm{with}\;\eta =\frac{\sigma_{m}}{\sigma_{vm}}$$

where $\epsilon_{p}$ is the accumulated plastic strain, $\epsilon_{f}\left(\eta \right)$ the failure strain as a function of stress triaxiality, and *n* is the damage exponent. Material instability is considered with the instability measure $F_{inst}$ defined by

(4) $$F_{inst}={\left(\frac{\epsilon_{p}}{\epsilon_{inst}\left(\eta \right)}\right)}^{n}.$$

**Figure A2:**
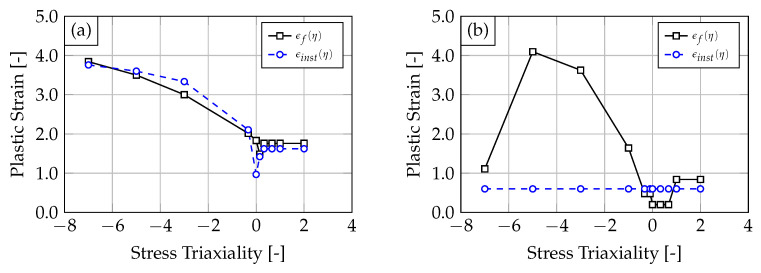
Implemented failure and instability parameters of GISSMO for the materials PA12 and PA6GF30 based on [15]: (**a**) PA12. (**b**) PA6GF30.

When $F_{inst}$ reaches unity, material damage takes place and is coupled with the stress tensor. Both the failure and instability strain as well as the damage exponent can be given as input for the GISSMO model. The damage model parameters were optimized prior to this investigation using quasi-static experiments [15]. In order to adapt the GISSMO model to the dynamic SHPB simulation, the quasi-static instability $\epsilon_{inst}\left(\eta \right)$ and failure strain $\epsilon_{f}\left(\eta \right)$ (see Appendix A Figure A2) were scaled depending on the strain rate. Modifications of the original failure and instability curves depending on the stress triaxiality were not made. The strain rate scale factor *r* was optimized using the SHPB simulations with indenter geometries V1, V4 and V5 in order to fit the experimental force–displacement relationship. As the literature values covering failure strain at different strain rates in compression are scarce, the scale factor *r* was determined at a fixed strain rate of 1000/s and interpolation was used between $r_{0}=1$ at quasi-static strain rate and the dynamic value. To avoid the solver-internal extrapolation to a scale factor of zero for even higher strain rates, a plateau with the value of r was used at strain rates above 1000/s.

## 3. Results and Discussion

### 3.1. Experimental

**Figure 7:**
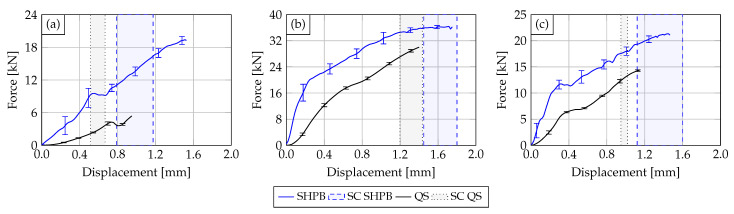
Comparison of the experimental results obtained from the quasi-static (solid black curve) and the dynamic characterization tests (solid blue curve) on the PA12 busbars with impactor V1 (**a**), V4 (**b**) and V5 (**c**). The according electrical SR ranges are also displayed.

In the following, the results of the conducted dynamic characterization tests (solid blue line) with indenter geometries V1, V4 and V5 are compared against quasi-static characterization tests (solid black line) obtained from Werling et al. [12]. The comparison is made in the form of force–displacement diagrams using the calculated mean value curves. Each mean value curve, which is based on the mean gradient change of the single test curves, was derived from five samples. Scattering bands display the single standard deviation of the derived force-displacement characteristics. The deformation areas in which short-circuits occur are delimited by dashed lines and highlighted in color. Figure 7 shows a comparison of the tested PA12 Cu-OFE R200 HVBs for each impactor configuration. It can be stated that the quasi-static and the dynamic mean curves show similar progressions in a qualitative sense and an overall rather ductile material behavior. However, the dynamic mean curves show a much stiffer behavior with an increase between 154% and 184% depending on the impactor type. Furthermore, the force level of the dynamic tests is significantly higher than in the quasi-static ones but shows a greater deviation. Additionally, the electric short circuits in the dynamic tests occurred in a higher displacement range.

**Figure 8:**
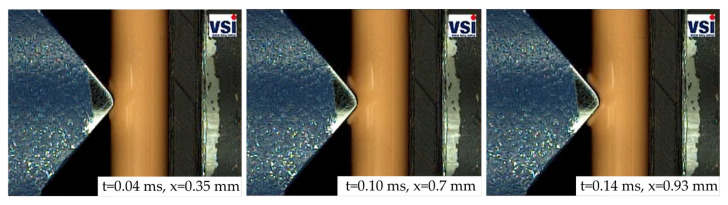
Exemplary high-speed sequences from a dynamic SHPB test on a PA12 busbar in combination with impactor V1.

The deformation behavior of the HVBs is exemplarily visualized in the form of high-speed sequences for impactor V1 (see Figure 8). In these three frames, the increase in plastic deformation of the insulation layer can be observed. Information about the current time step (t) and the current displacement (x) is also given for each frame. Typical for the ductile PA12 insulation layer is the material pile-up at the indenter edges (see Figure 8 x=0.93 mm). No crack initiation was observed.

**Figure 9:**
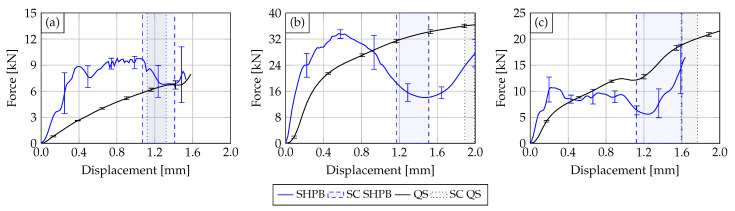
Comparison of the experimental results obtained from the quasi-static (solid black curve) and the dynamic characterization tests (solid blue curve) on the PA6GF30 busbars with impactor V1 (**a**), V4 (**b**) and V5 (**c**). The according electrical SR ranges are also displayed.

**Figure 10:**
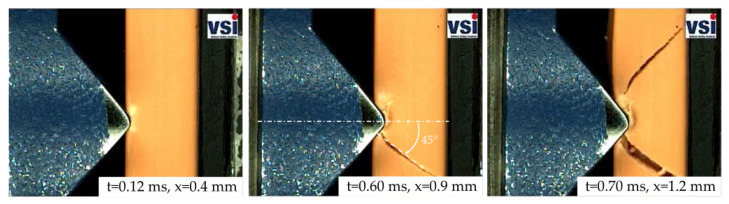
Exemplary high-speed sequences from a dynamic SHPB test on a PA6GF30 busbar in combination with impactor V1.

In the case of the tested PA6GF30 Cu-OFE R240 HVBs, the comparison between the quasi-static and dynamic mean curves yields a significant difference. While the quasi-static material response is relatively ductile, the dynamic one is quite brittle. This can be seen in the massive force drops in the rear half of the dynamic curves (see Figure 9), which are related to the crack growth in the insulation (see Figure 10). It is assumed that the brittle failure of the insulation material in the dynamic tests is responsible for the early occurrence of the short circuit compared to the quasi-static tests. Similar to the PA12 Cu-OFE R200 HVBs, the PA6GF30 Cu-OFE R240 HVBs also show a higher initial stiffness (+96% up to +249% depending on the impactor type) and a higher single standard deviation in the dynamic tests.

The crack initiation and crack growth behavior of the relatively brittle PA6GF30 insulation can be seen in the high speed sequences in Figure 10. The cracks form at an early stage of the deformation process with a 45° angle with respect to the loading direction.

**Figure 11:**
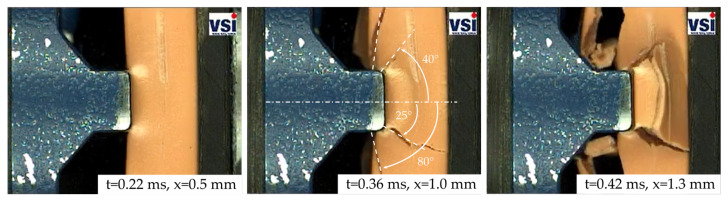
Exemplary high-speed sequences from a dynamic SHPB test on a PA6GF30 busbar in combination with impactor V4.

Contrary to the crack-formation with impactor V1, the situation with impactor V4 is in some way different (see Figure 11). Here, four crack paths were forming almost simultaneously. Two of these crack paths show angles between 25° and 40° while the other two are running at roughly 80° with respect to the loading direction. All four cracks were initiated at the edges of the impactor.

A comparison of the critical force, penetration and energy values between the quasi-static and the dynamic experiments is given in Table 2. The force and penetration data were evaluated at the mean occurrence of the short circuit (SC) in terms of displacement whereas “energy” stands for the absorbed deformation energy to critical mean penetration (to mean SC) of the impactor. It can be concluded that PA12 Cu-OFE R200 HVBs can take up significantly more deformation energy to failure at high strain rates. On the other hand, the situation for the PA6GF30 Cu-OFE R240 busbars is quite different, which is due to the mostly brittle failure characteristic at high strain rates. In two (V4 and V5) out of three cases, the force penetration and energy level was significantly lower than in the quasi-static tests.

### 3.2. Numerical

**Figure 12:**
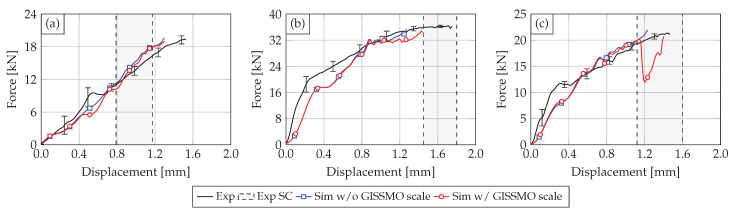
Comparison of the experimental (solid black curve) and numerical results without strain rate scaling GISSMO parameters (solid blue curve) and with strain rate scaling (solid red curve) for the PA12 busbars with impactor V1 (**a**), V4 (**b**) and V5 (**c**). The experimental electrical SR ranges are also displayed.

The numerical results of the SHPB simulation with the PA6GF30 Cu-OFE R240 and the PA12 Cu-OFE R200 busbars are presented in the following. A comparison is made between the results using the quasi-static GISSMO parameters and the results depending on the strain rate. The results for the PA12 insulated busbars are shown in Figure 12. The underlying scale factor for the failure and instability strain of the GISSMO model was set to r=0.54 at a strain rate of 1000/s.

The results for impactor geometry V1 are displayed in Figure 12a. It can be seen that the high strain rate response of PA12 is not governed by material failure or damage as the simulation with and without GISSMO scale shows almost identical behavior. No element failure occurs in the simulation with the quasi-statically defined GISSMO parameters. However, using the GISSMO strain rate scaling, few elements fail starting at intrusions of 0.4 mm and therefore reducing the reaction force. The failed elements are located beneath the impactor tip. In the range where electrical SC occurs, the simulation shows good agreement with the experiment. At the earliest SC in terms of impactor intrusion at 0.8 mm, the force with GISSMO scale differs only 5% from the experiment (without scale factor 2%).

The results for the impactor geometries V4 and V5, displayed in Figure 12b,c, show similar results. The simulations with and without GISSMO scale differ only with the onset of material failure. While both simulations underestimate the materials stiffness up to displacements of 0.8 mm (V4) and 0.5 mm (V5), a good accuracy in the range of experimental deviations is achieved for larger displacements.

The difference between the experimental and numerical material stiffness at smaller displacements can be explained by the lack of visco-elasticity, common for thermoplastics, in the used material models. Another aspect of the deviation between experiment and simulation lies in the initial slope of the curves (first 0.1 mm).

Here, the simulation shows a softer initial behavior compared to the experiments. After this initial slope, both the experiment and simulation show almost identical stiffness properties. At the earliest electrical SC at 1.45 mm for impactor V4, the experimental and numerical force differ by 4%. For impactor V5, an even better agreement between experiment and simulation was achieved, as the deviation at a displacement of 1.12 mm is only 3%. It is notable that the force drop in the simulation due to the sudden failure of adjacent elements convinced with the earliest SC for impactor V5.

**Figure 13:**
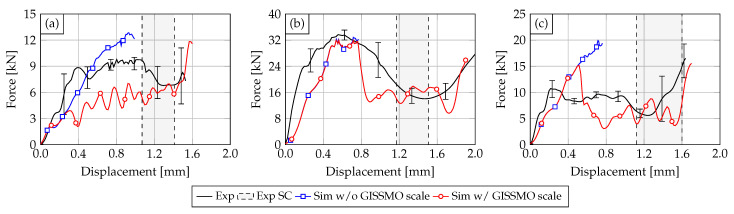
Comparison of the experimental (solid black curve) and numerical results without strain rate scaling GISSMO parameters (solid blue curve) and with strain rate scaling (solid red curve) for the PA6GF30 busbars with impactor V1 (**a**), V4 (**b**) and V5 (**c**). The experimental electrical SR ranges are also displayed.

The results for the PA6GF30 Cu-OFE R240 busbars are shown in Figure 13. Here, the strain rate scale factor for GISSMO was set to 0.22 at a strain rate of 1000/s and a linear decrease with increasing strain rate was assumed. As the PA6GF30 material behavior is governed by brittle failure and crack development, it can be noted that the overall accuracy of the simulation reduces compared to the ductile PA12 insulated busbars. Furthermore, the initial material stiffness is underestimated for all impactor geometries. Here, a lack of visco-elasticity in the material model is more significant compared to the simulation with PA12.

**Figure 14:**
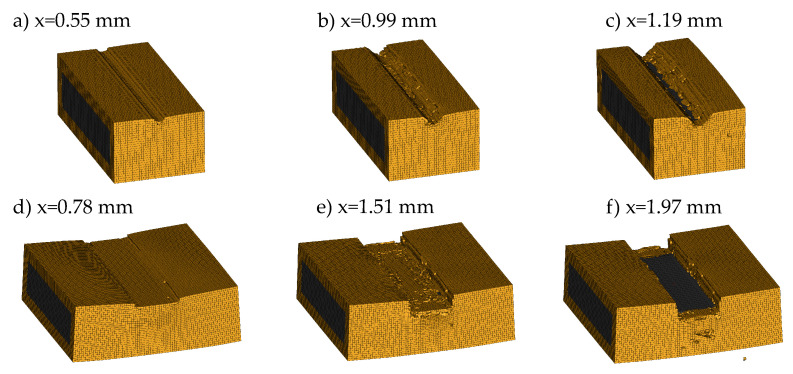
Exemplary sequences from the SHPB simulation on a PA6GF30 insulated busbar in combination with impactor V1 and V4 using the GISSMO strain rate scale factor: (**a**–**c**) show impactor V1 and (**d**–**f**) show impactor V4.

For impactor geometry V1, no improvement could be achieved by strain rate scaling of the GISSMO parameters. While no element failure occurs without the scale factor, hence, overestimating the material strength, continuous element deletion starting at a displacement of 0.35 mm reduces the reaction force significantly. At the earliest SC at 0.79 mm, the force differs by 29% compared to the experiment. For displacements around 1.2 mm, the numerical result with GISSMO scale approximates the experimental average and is within the test deviations. Figure 14a–c displays the simulation result for indenter V1 at different stages of the deformation. It can be seen that element failure is limited to the area below the impactor, but no crack-formation similar to the experiment can be observed.

The results for impactor V4 are displayed in Figure 13b. The simulation without the GISSMO scale reaches a maximum impactor displacement of 0.78 mm. Around this displacement, material failure takes place in the simulation with the strain rate scaling leading to a sudden force drop. Subsequently, the simulation force fluctuates around 16 kN due to continuous element deletion until a displacement of 1.7 mm is reached. At this stage, the impactor also contacts the copper material of the busbar. The qualitative mechanical response of the simulation matches with the experimental average and at the earliest SC, the simulation force differs by 14% compared to the experiment. The deformed busbar FE model at different stages of the deformation is displayed in Figure 14d–f. Although brittle material behavior is present, no crack-formation is visible. Instead, elements fail continuously below the impactor and at the busbar edges until the copper material below is contacted.

The behavior of the busbar subjected to impactor V5 in Figure 13c is similar to V4. Following a significant force drop at the onset of material failure, the simulation force with the strain rate scaling fluctuates around 6 kN until the copper is hit by the impactor. In contrast to V4, the maximum force is higher than in the experiment reaching 11 kN. At the earliest SC, the force differs by 15% compared to the experimental average.

## 4. Conclusions

In this study, the electro-mechanical behavior of HVBs under high-loading rates, as they might occur in a vehicle crash, were investigated using an SHPB.

The results obtained in the high-loading-rate experiments shed a new light on the behavior of the isolation, which was not anticipated based on the previous low-loading-rate experiments. It was found that the PA6GF30 insulated HVB performed subpar in dynamic loading, and showed a more brittle behavior and earlier failure than in quasi-static loading. In comparison, the PA12 Cu-OFE R240 busbar showed a significant increase in critical values compared to quasi-static tests until electrical failure in the form of a short circuit occurred. The phenomenological behavior of PA12 was similar to the quasi-static observed one [12].

One major outcome of the numerical modeling approach was that, using the sophisticated GISSMO damage model, a good qualitative and quantitative agreement with the experimental data was achieved for PA12 and the two blunt impactors for the PA6GF30 busbar. However, for the sharp impactor in combination with the PA6GF30 busbar, the numerical model performed sub-optimally. The simulations showed that extreme brittle material failure of PA6GF30 in the tested loading scenario could not be reproduced phenomenologically using the damage and failure model.

The methodology presented in this research was helpful to study the behavior of HVB insulation materials under high strain rates and is deemed useful for the testing and validation of other high-voltage components in the future. Future work will cover the improvement of the material and failure models. Here, the implementation of visco-elasticity as well as the strain rate dependent modification of failure and instability functions will be investigated.

## Figures and Tables

**Table 1:** Material properties of the investigated busbars extracted from data sheets for copper [21] and polyamide [22,23]. The properties of PA12 and PA6GF30 are given at a conditioned state (23 °C and 50% relative humidity).

Property		PA12	PA6GF30	Cu-OFE R200	Cu-OFE R240
Density	(g/cm^3^)	1.01	1.39	8.94	8.94
Young’s Modulus	(GPa)	1.2	4.5	127	127
Yield Stress	(MPa)	40	60	<140	>180
Tensile Strength	(MPa)	-	-	200–260	240–300
Melting Point	(°C)	178	222	1084	1084
Moisture Absorption	(%)	0.7	2.5	-	-

**Table 2:** Comparison of force, penetration and energy values between the quasi-static (q.s.) and the dynamic (dyn.) experiments for the impactor types V1, V4 and V5.

Material	Indenter	Force (kN)			Penetration (mm)			Energy (J)		
		q.s.	dyn.	Δ	q.s.	dyn.	Δ	q.s.	dyn.	Δ
	V1	3.47	13.5	+289%	0.601	0.941	+56.7%	0.60	6.52	+987%
PA12	V4	29.9	36.3	+21.4%	1.286	1.613	+25.5%	20.0	43.9	+119%
	V5	14.1	20.7	+46.5%	0.996	1.310	+36.6%	6.55	18.8	+187%
	V1	6.68	7.06	+5.7%	1.223	1.264	+3.32%	4.35	9.30	+113%
PA6GF30	V4	35.0	15.4	−56.0%	1.955	1.346	−31.1%	50.5	33.1	−34.5%
	V5	19.4	6.50	−66.5%	1.657	1.390	−16.1%	17.8	11.0	−38.2%

## Data Availability

Data obtained is contained within the article.

## Abbreviations

The following abbreviations are used in this manuscript:

HVB	High-voltage busbar
SHPB	Split Hopkinson pressure bar
SC	Short circuit
FE	Finite element
PLA	Polylactic acid
QS	Quasi-static
